# A Novel Strategy for Detection and Enumeration of Circulating Rare Cell Populations in Metastatic Cancer Patients Using Automated Microfluidic Filtration and Multiplex Immunoassay

**DOI:** 10.1371/journal.pone.0141166

**Published:** 2015-10-23

**Authors:** Mark Jesus M. Magbanua, Michael Pugia, Jin Sun Lee, Marc Jabon, Victoria Wang, Matthew Gubens, Karen Marfurt, Julia Pence, Harwinder Sidhu, Arejas Uzgiris, Hope S. Rugo, John W. Park

**Affiliations:** 1 Division of Hematology-Oncology, University of California San Francisco, San Francisco, CA, 94115, United States of America; 2 Siemens Healthcare Diagnostics, Elkhart, IN, 46516, United States of America; Northwestern University Feinberg School of Medicine, UNITED STATES

## Abstract

Size selection via filtration offers an antigen-independent approach for the enrichment of rare cell populations in blood of cancer patients. We evaluated the performance of a novel approach for multiplex rare cell detection in blood samples from metastatic breast (n = 19) and lung cancer patients (n = 21), and healthy controls (n = 30) using an automated microfluidic filtration and multiplex immunoassay strategy. Captured cells were enumerated after sequential staining for specific markers to identify circulating tumor cells (CTCs), circulating mesenchymal cells (CMCs), putative circulating stem cells (CSCs), and circulating endothelial cells (CECs). Preclinical validation experiments using cancer cells spiked into healthy blood demonstrated high recovery rate (mean = 85%) and reproducibility of the assay. In clinical studies, CTCs and CMCs were detected in 35% and 58% of cancer patients, respectively, and were largely absent from healthy controls (3%, *p* = 0.001). Mean levels of CTCs were significantly higher in breast than in lung cancer patients (*p* = 0.03). Fifty-three percent (53%) of cancer patients harbored putative CSCs, while none were detectable in healthy controls (*p*<0.0001). In contrast, CECs were observed in both cancer and control groups. Direct comparison of CellSearch^®^ vs. our microfluidic filter method revealed moderate correlation (R^2^ = 0.46, kappa = 0.47). Serial blood analysis in breast cancer patients demonstrated the feasibility of monitoring circulating rare cell populations over time. Simultaneous assessment of CTCs, CMCs, CSCs and CECs may provide new tools to study mechanisms of disease progression and treatment response/resistance.

## Introduction

Recent technological advances have enabled the reliable detection and characterization of circulating tumor cells (CTCs) in the blood of cancer patients [1, 2]. To quantify levels of CTCs, assays have been developed to facilitate the detection of epithelial cells in the blood by using cellular markers such as EPCAM and cytokeratins [3]. Consequently, the clinical significance of these cells has been demonstrated in numerous studies showing that elevated CTC numbers are associated with reduced survival and poor response to therapy [4–7]. However, recent studies have identified CTCs with low expression of epithelial markers, e.g., those undergoing epithelial-mesenchymal transition (EMT), which may not be detected by the epithelial-based assays [8, 9]. Antigen-independent enrichment, such as filtration systems, offers an alternative approach that can potentially eliminate selection bias due to reliance on specific antigen expression [10–14]. Filter-based methods facilitate the enrichment of non-hematologic rare cells (including CTCs) by exploiting the size differences between these cells and cells in peripheral blood [10–14]. While leukocytes and erythrocytes typically measure about 6 to 8μM in diameter, CTCs can vary in size, with diameters of 10μM or greater [15, 16]. Finally, cells that are captured on the filter can be subjected to further assay for detection and enumeration of CTCs [17–19].

In this study, we evaluated the performance of a novel approach for detection and enumeration of multiple rare cell populations in the blood of metastatic breast and lung cancer patients using an automated microfluidic filtration and multiplex immunoassay strategy. Different circulating rare cell populations were detected and enumerated, including circulating tumor cells (CTCs), circulating mesenchymal cells (CMCs), circulating endothelial cells (CECs), and putative circulating stem cells (CSCs). CTC counts were compared to results from the CellSearch^®^ system, a U.S. Food and Drug Administration (FDA)-cleared assay for enumeration of CTCs. We also tested the feasibility of serial blood analysis in a subset of breast cancer patients.

## Methods

### Ethics Statement

This study was approved by the Institutional Review Boards at the University of California San Francisco (UCSF; Committee on Human Research) and Siemens Healthcare Diagnostics (Elkhart, IN; Schulman Associates IRB). Informed written consent was obtained from each cancer patient and healthy volunteer who participated in this study. Cancer patients were enrolled between January and August of 2014 at UCSF. Healthy donors were recruited at Siemens.

### Patient and Healthy Blood Sample Collection

Approximately 6-9mL of blood was collected into tubes containing potassium ethylenediamine tetraacetic acid (K_3_EDTA) and 0.45mL Transfix^®^ (Vacutest Kima). Samples from UCSF were shipped to Siemens Healthcare Diagnostics in insulated boxes equipped with controlled room temperature packs (Saf-T-Pak™ Inc.) and a digital temperature recorder (Track-It™, Monarch Instrument). Blood samples were stored at ambient laboratory temperature until further processing.

### Control Preparation

Positive and negative controls were prepared using blood samples from healthy donors. Approximately 8mL of blood was drawn into tubes containing K_3_EDTA and 0.045% Transfix^®^. For positive controls, 10μL of fixed cultured cells (at 10^5^ cells/mL)—either SKBR3 (a breast cancer line, American Type Culture Collection ATCC^®^ HTB-30^TM^) or NCI H226 (a lung cancer line, ATCC^®^ CRL-5826^TM^)—were spiked into healthy blood. A positive control containing approximately 1000 cells and a negative control (non-spiked blood from healthy donor) were included in each run.

### Circulating Cell Isolation and Immunocytochemistry Staining

All blood samples were processed at Siemens Healthcare Diagnostics. Processing steps that included filtration, cell isolation, and immunocytochemistry (ICC) staining were performed using a Hamilton STARlet™ robot (Hamilton Company, Reno Nevada). The instrument was specially equipped with a filtration device and software to control the robotic automation of downstream processes. A filter membrane with surface area of 3.8cm^2^ with pore size of 8μm (Whatman™ Nuclepore™, GE Healthcare) was fitted onto the device. The 8μm pore size was selected based on optimal cell recovery as reported in the literature [15, 20, 21]. Details of the filtration and cell isolation procedures are described in S1 File and S1 Fig. Briefly, whole blood samples were diluted with phosphate buffered saline (PBS) containing 0.083% fibrinogen. The diluted samples were then filtered through the microfluidic filter device. This process enriches for circulating rare cells but also retains approximately 50,000–100,000 white blood cells (WBCs). The cells captured on the filter membrane were fixed, and automated ICC staining was performed to identify specific cell populations. Optimization of assay parameters is discussed in S2 File.

S1 Table lists the antibodies and fluorescent probes used in this study. Details of the ICC procedure are described in S1 File. Briefly, cells were permeabilized, and a blocking solution was added to prevent non-specific binding of antibodies. Cells were incubated in an appropriate antibody solution for staining of target antigens. A flow chart illustrating the staining process is shown in S1 Fig Optimization of assay parameters is discussed in S2 File.

The following cells lines were used as controls to determine thresholds for positive immunostaining signals: (1) SKBR3, a breast cancer cell line that is CK+, VIM-, CD144-, TPBG/5T4-, PIWIL2-, CD144-; (2) NCI-H2228, a lung cancer cell line that is CK+, VIM+, CD144-, TPBG/5T4+, PIWIL2-, CD144-; (3) NCI-H266, a lung cancer line that is CK+, VIM+, TPBG/5T4-, PIWIL2-, CD144-; (4) MDA-MB-231, a breast cancer cell line that is CK+, VIM-, TPBG/5T4-, PIWIL2-, CD144-; and (5) a transfected MDA-MB-231 expressing PIWIL2 and therefore is CK+, VIM-, TPBG/5T4-, PIWIL2+, CD144 (gift of Dr. Jian-Xin Gao, Shanghai Jiao Tong University School of Medicine, Shanghai, China). Normal leukocytes and endothelial cells were used as positive controls for CD45 and CD144 staining, respectively, and as negative controls for all other markers.

### Microscopic Analysis and Enumeration of Circulating Rare Cells

Detection and imaging of cells were performed using a fluorescence microscope (Leica DM5000, Leica Microsystems GmbH) (S1 File). Different cell types were enumerated based on staining patterns observed.

The initial detection threshold was defined as the point at which the fluorescence signal was detectable to be higher than non-specific fluorescence from the filtration membrane. Analytical cut-offs were further defined based on fluorescence levels of known cancer cells (SKBR-3 or H226) and WBCs from healthy controls utilized as controls for positive and negative signals, respectively. For consistency, only one investigator (KM) counted the cells, while another investigator (MP) confirmed the cell counts.

### Statistical Analysis

We analyzed enumeration results (cells/mL) using t-tests. The Fisher’s exact test was used to compare the detection rate between groups. A *p*-value <0.05 was considered statistically significant.

## Results

### Assay Design

**Fig 1:**
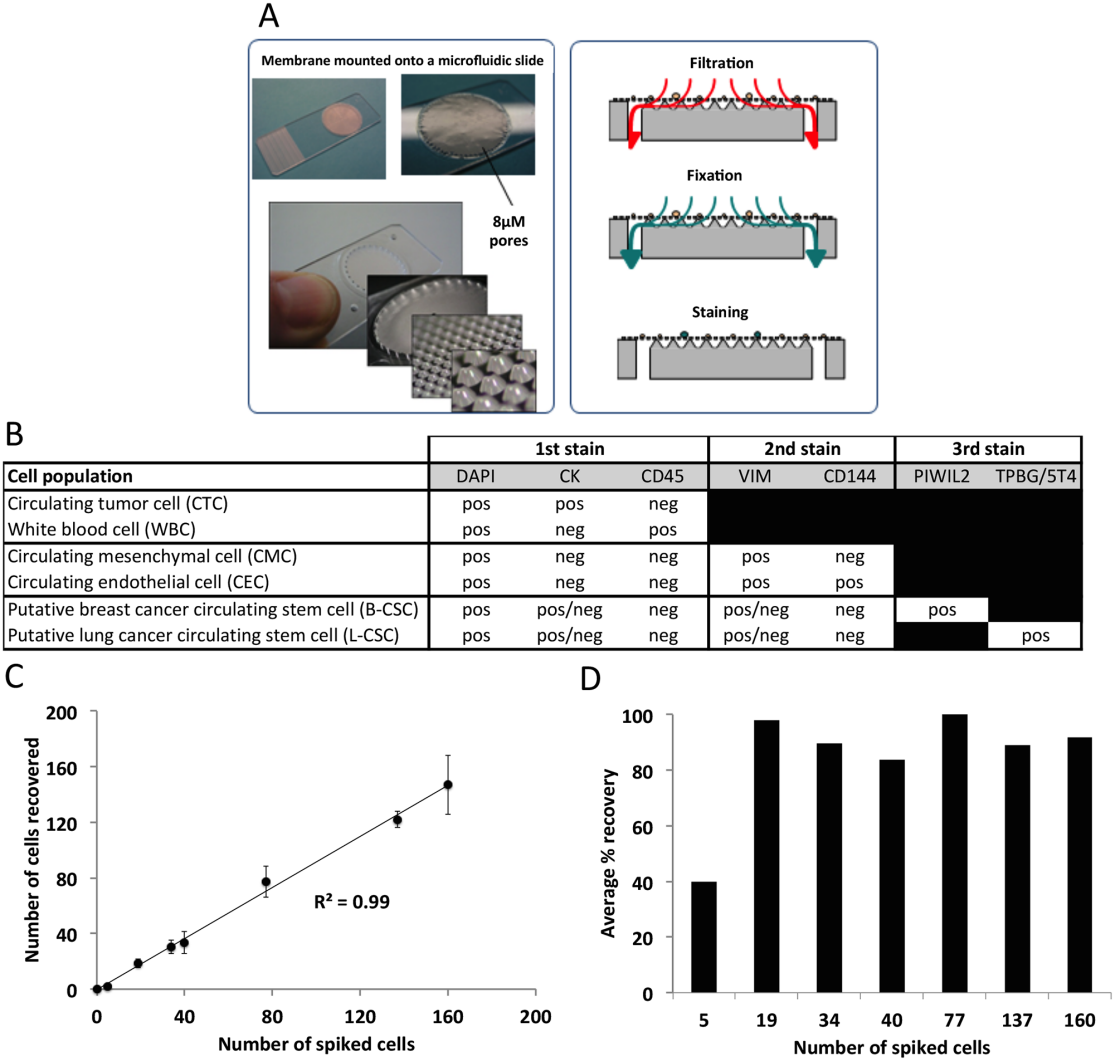
Assay development and cell recovery experiment. **A.** Design of the microfluidic filter device and the schematic overview of the processing steps for capture and detection of circulating rare cells, **B**. Biomarkers and immuno-phenotypes of circulating rare cells identified through the sequential staining procedure, **C.** Average percent recovery after spiking known numbers of H226 lung cancer cells into healthy blood, **D.** Scatter plot showing the number of H226 spiked cells and the number of cells recovered.

We evaluated a novel strategy for multiplex rare cell enumeration using an automated filtration-based instrument (Siemens Healthcare Diagnostics) and staining with multiple immunofluorescence cocktails. The device consists of a filter membrane mounted to a micromachine support that allows isolation of rare cells, followed by autostaining and microscopic detection. This disposable integrated cell capture and characterization device etched with microfluidic structures allows for controlled filtration across a pressure range of 10–30 mbar (Fig 1A). Detailed design of the microfluidic device is shown in S3 Fig.

The CTC staining cocktail included 4',6-diamidino-2-phenylindole (DAPI), a pan-cytokeratin (pan-CK) antibody, and antibodies to CK8/18, CK19, and CD45 (S1 Table). DAPI fluorescence was detected in the blue channel and was used to identify nucleated cells. Fluorescent Pan-CK, CK8/18, and CK19 antibodies were used to label cells of epithelial origin and were detected in the red channel, while fluorescent antibodies to CD45 were used to label cells of hematopoietic origin and were detected in the far-red channel. CTCs were defined as nucleated, CK-positive, and CD45-negative, while WBCs were defined as nucleated but were CK-negative and CD45-positive (Fig 1B).

The CMC/CEC staining cocktail included antibodies for vimentin (VIM) and CD144. The VIM marker was used to identify cells undergoing EMT. Cells labeled with fluorescent antibodies to VIM were detected in the red channel. Antibodies to CD144 were used to identify cells of endothelial origin. Labeled cells were detected in the green channel. Also, the distinctive staining pattern for CD144 demonstrating localization on the cell membrane facilitated the identification of CECs. The results of this staining process were combined with those from the initial stain, as the DAPI and the CD45 signals were still visible. CMCs were defined as nucleated, VIM-positive, CK-negative, CD144-negative, and CD45-negative. CECs were defined as nucleated, CD144-positive, VIM-positive, CK-negative, and CD45-negative.

The CSC staining process utilized a high sensitivity immunoassay method using Tyramide Signal Amplification (TSA™, Life Technologies) to detect the expression of candidate stem cell markers, PIWIL2 and TPBG/5T4, which are expressed at low levels. Amplified signals were detected in the green channel and were used to identify putative CSCs defined as nucleated, stem cell marker-positive, CK-positive/negative, VIM-positive/negative, CD144-negative, and CD45-negative. TPBG/5T4 expression was used to identify putative lung cancer CSCs while PIWIL2 expression was used to detect putative breast cancer CSCs.

The highly controlled filtration process and the multi-step staining parameters were optimized to minimize the detection of false positives in healthy donor blood. Use of a proprietary blood collection tube containing an optimized fixative formulation (TransFix^®^), along with controlled shipping and storage conditions contributed to the high rate of reportable results (98%).

### Analytical Performance

First, we evaluated the performance of the microfluidic filter device using a spike-in model involving H226 lung cancer cells. A range of 5–160 cells was spiked into healthy donor blood and isolated using the device. ICC staining and microscopic analyses were performed to detect and enumerate nucleated, CK-positive, and CD45-negative cells. Uniformity of cell counts observed among triplicate samples indicated high reproducibility (Fig 1C). In addition, we observed a high correlation between observed and expected yield (R^2^ = 0.99). Isolation of spiked cells was very efficient, with an overall recovery rate of 85% (Fig 1D). With 5 spiked cells, lower recovery of 40% was observed, and was attributable to tube adherence and cell loss. These results are entirely comparable with those of CellSearch [22, 23]. For example, CellSearch enumeration of spiked samples was 80% and 82% [22]. Allard and colleagues [23] reported recovery with a 4 cell spike showed standard deviation of 2 and 95% confidence interval of 1–11, which are statistically indistinguishable from our results with 5 cell spike.

### Clinical Testing

**Fig 2:**
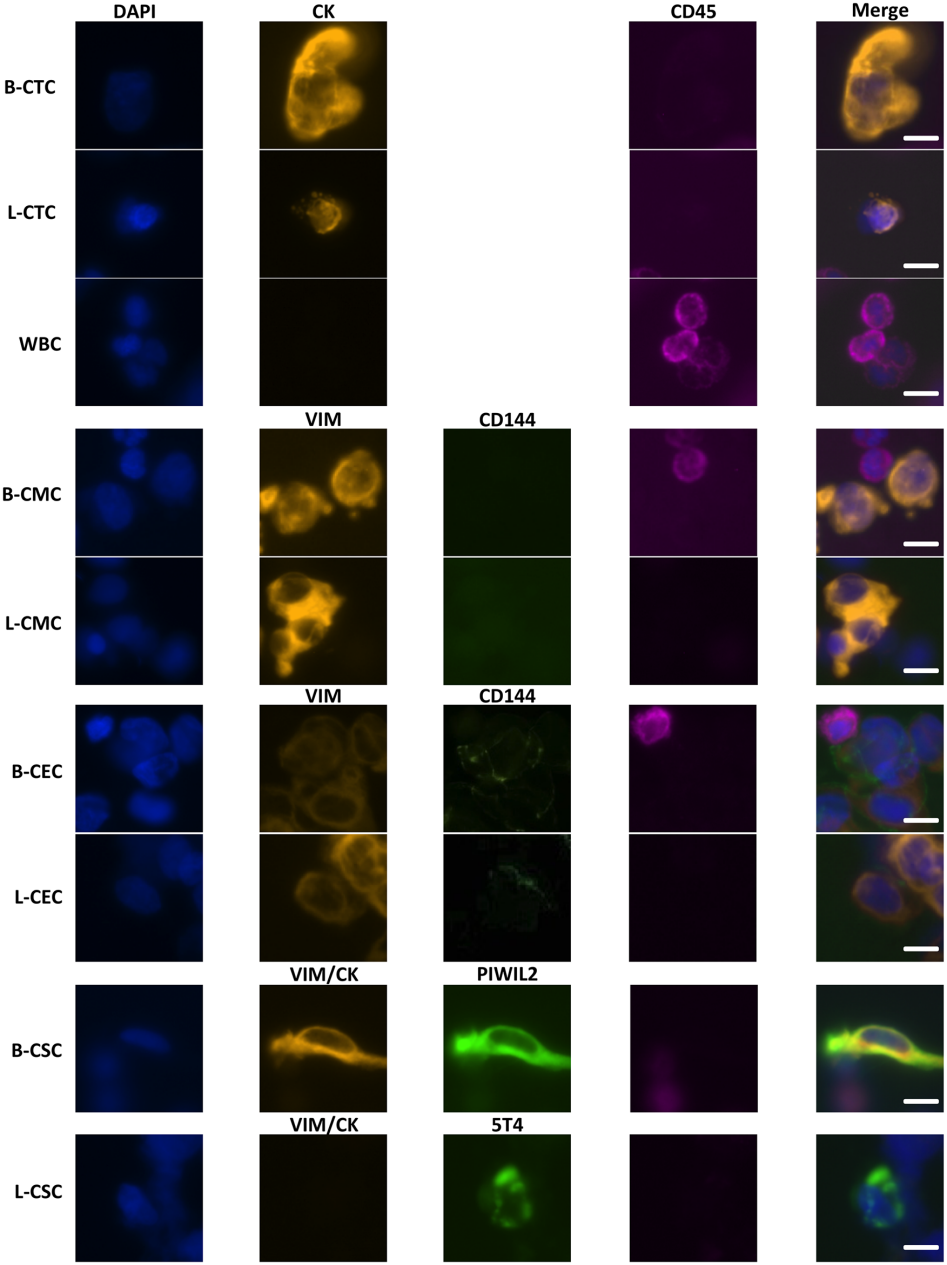
Immunocytochemistry staining of circulating rare cells. Representative images of circulating tumor cells (CTC), white blood cells (WBC), circulating mesenchymal cells (CMC), circulating endothelial cells (CEC), and putative circulating stem cells (CSC) in metastatic breast (B) and lung (L) cancer patients. The scale bar represents 8μM.

Clinical validation of the microfluidic filter device was performed on whole blood samples from metastatic breast and lung cancer patients. To determine background levels in healthy individuals, blood samples from 30 controls were also evaluated. Samples were processed via the microfluidic filter device, and cells captured on the membrane were stained for markers to identify circulating cells of interest. Representative images of different cell types are illustrated in Fig 2.

Patient characteristics are presented in Table 1. Of the 43 patients enrolled, 40 were evaluable (S2 Fig). The final cohort was composed of 19 breast cancer patients and 21 lung cancer patients. Of the 19 breast cancer patients, 95% were ER-positive and 26% were HER2-positive, while 81% and 19% of the lung cancer patients were diagnosed with non-small cell lung carcinoma (NSCLC) and small cell lung carcinoma (SCLC), respectively. Clinically relevant driver mutations, e.g., in *EGFR*, *KRAS*, and *ELM4-ALK* fusion, were present in 40% of the lung cancer patients.

**Table 1:** Patient characteristics. Abbreviations: ER-estrogen receptor, NSCLC- non-small cell lung cancer, SCLC- small cell lung cancer.

Breast cancer (n = 19)	No.	%*
ER status		
ER-positive	18	95
ER-negative	1	5
HER2 status		
HER2-positive	5	26
HER2-negative	13	68
Unknown	1	5
Site of metastasis		
Bone	14	74
Other	5	26
**Lung cancer (n = 21)**		
Histology		
NSCLC-Adenocarcinoma	14	67
NSCLC-Squamous	2	10
NSCLC-Large cell neuroendocrine	1	5
SCLC	4	19
Genes with relevant mutations		
*EGFR*	1	5
*EML4-ALK fusion*	2	10
*KRAS*	2	10
*BRAF*	1	5
*SETD2*	1	5
*ROS1*	1	5
No mutation detected	9	43
Not tested	4	19
Site of metastasis		
Brain	4	19
Other	16	76
No data	1	5

*Percentages have been rounded and may not total to 100%.

### Detection and Enumeration of Rare Circulating Cell Types

We analyzed staining patterns on captured cells using fluorescence microscopy to enumerate CTCs, CMCs, CECs, and putative CSCs in blood of cancer patients and healthy controls. Enumeration results are summarized in Table 2.

**Table 2:** Enumeration of circulating rare cells. Abbreviations: Circulating tumor cells (CTC), circulating mesenchymal cells (CMC), circulating endothelial cells (CEC) and putative circulating stem cells (CSC).

All cancer patients (n = 40)	CTC/mL	CMC/mL	CTC&CMC/mL	CSC/mL	CEC/mL
**Mean**	0.22	1.40	1.62	0.16	3.61
**SD**	0.49	5.46	5.45	0.30	8.01
**Median**	0.00	0.13	0.31	0.12	0.13
**Range**	0–2.29	0–34.00	0–34.00	0–1.64	0–41.09
**Breast cancer (n = 19)**					
**Mean**	0.41	0.34	0.75	0.15	2.58
**SD**	0.66	0.50	0.88	0.21	4.62
**Median**	0	0.13	0.38	0.13	0.25
**Range**	0–2.29	0–1.83	0–2.86	0–0.83	0–17.09
**Lung cancer (n = 21)**					
**Mean**	0.05	2.37	2.41	0.16	4.54
**SD**	0.10	7.48	7.47	0.37	10.19
**Median**	0	0.13	2.63	0.06	0.12
**Range**	0–0.35	0–34.00	0–34.00	0–1.64	0–41.09
**Healthy controls (n = 30)**					
**Mean**	0	0	0	0	0.47
**SD**	0.02	0	0.02	0	1.31
**Median**	0	0	0	0	0
**Range**	0–0.11	na	0–0.11	na	0–7.11

**Fig 3:**
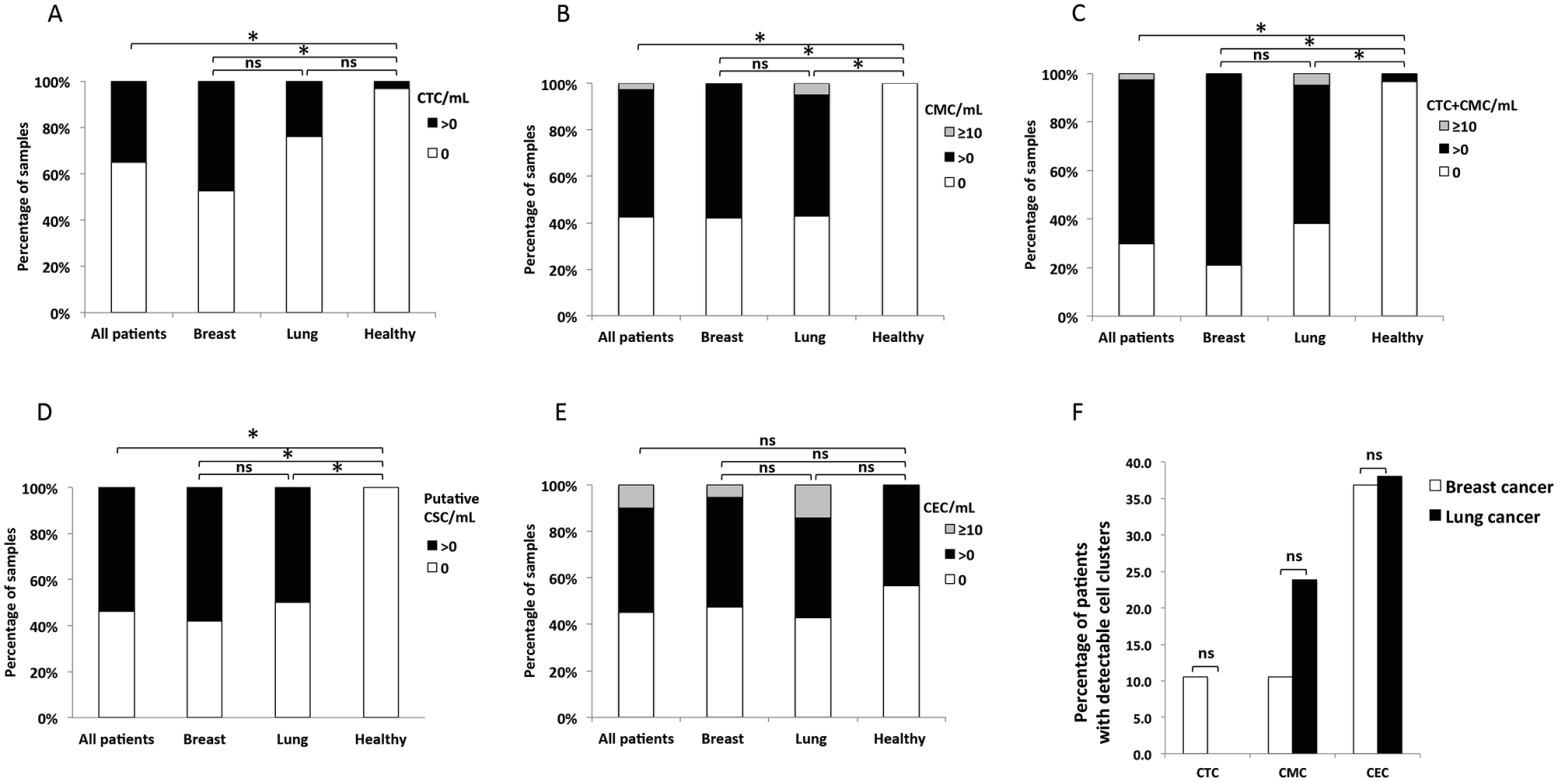
Detection of circulating rare cells in metastatic breast and lung cancer patients and healthy controls. Percentage of samples with detectable rare cells: **A.** circulating tumor cells (CTC), **B.** circulating mesenchymal cells (CMC), **C.** CTC and CMC, **B.** circulating mesenchymal cells (CMC), **D**. putative circulating stem cells (CSC), and **E.** circulating endothelial cells (CEC) in metastatic breast and lung cancer patients, and healthy controls. **F.** Percentage of patients with detectable cell clusters. Percent detection between groups was compared using Fisher exact tests and was considered significant (*) when *p*-value was <0.05, otherwise, not significant (ns).

**Fig 4:**
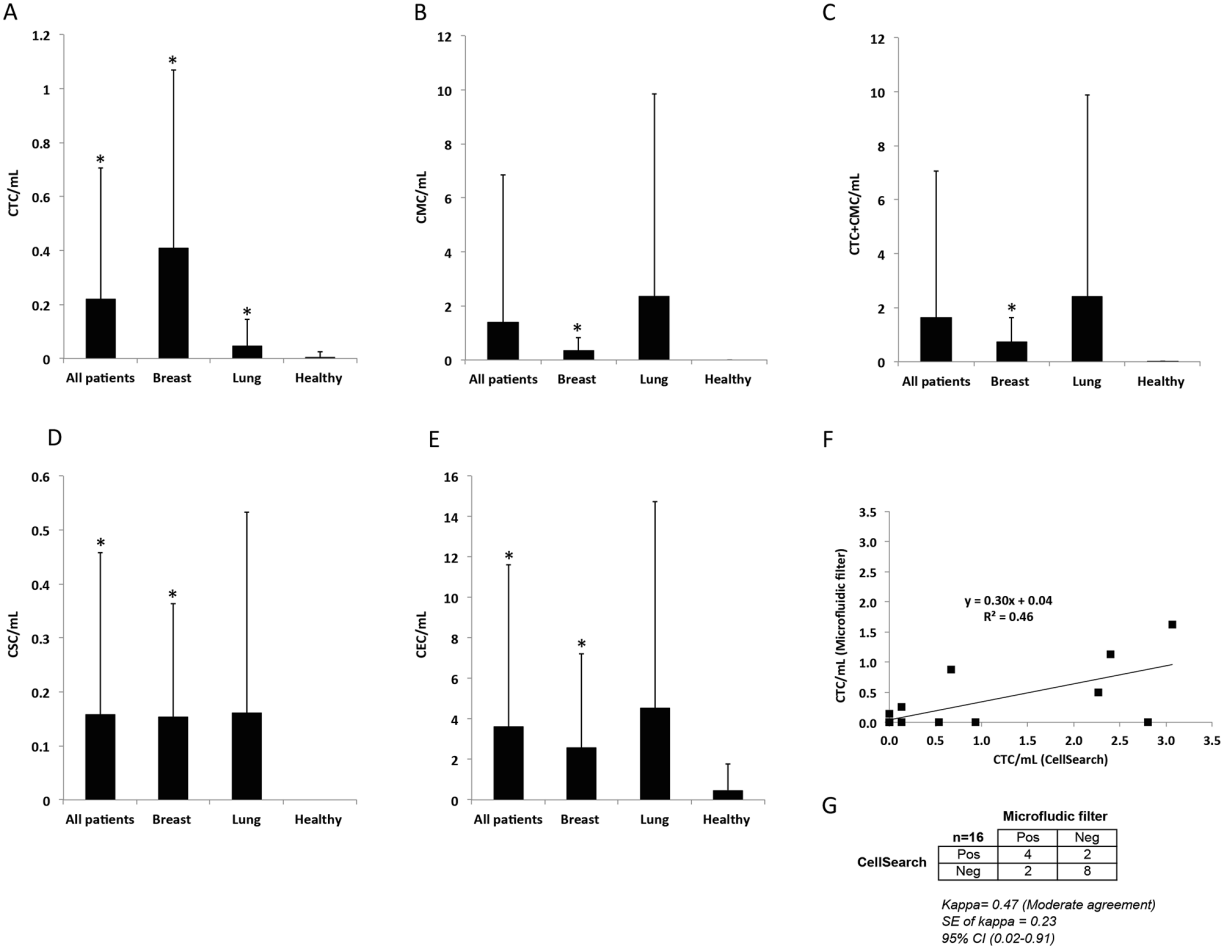
Enumeration of circulating rare cells in metastatic breast and lung cancer patients and healthy controls. Mean cells per mL for **A.** circulating tumor cells (CTC), **B.** circulating mesenchymal cells (CMC), **C.** CTC and CMC, **D.** putative circulating stem cells (CSC), and **E.** circulating endothelial cells (CEC) in metastatic breast and lung cancer patients and healthy controls. A single sample t-test was used to compare mean cells per mL to population mean of 0. An asterisk (*) indicates a *p*-value <0.05, **F.** Comparison of enumeration results between CellSearch^®^ and the microfluidic filter assay, **G.** Agreement in positive and negative calls between CellSearch^®^ and the microfluidic filter assay in 16 blood samples. Samples with ≥5 circulating tumor cells (CTC) per 7.5mL of blood were considered positive using the CellSearch^®^ assay while samples with detectable CTC (>0) were considered positive using the microfluidic filter-based assay.

CTCs were detected in 35% of cancer patients, including 47% of the breast cancer patients and 24% of the lung cancer patients (Fig 3A). CTCs were largely absent from controls, with the exception of a single cell detected in one healthy donor (3%). CTC detection in cancer patients was significantly higher than in healthy controls (*p*<0.001). The mean CTC level in breast cancer patients (0.41 CTC/mL) was significantly higher than in lung cancer patients (0.04 CTC/mL, *p* = 0.03) (Table 2, Fig 4A).

CMCs were detected in 58% of cancer patients, including 58% in breast cancer and 57% in lung cancer (Fig 3B). CMC detection was significantly higher in cancer patients than in healthy controls, in whom no CMCs were detected (*p*<0.001). Mean CMC levels were 0.34 CMC/mL in breast cancer patients and 2.37 CMC/mL in lung cancer patients, which was not a significant difference (*p* = 0.23) (Table 2, Fig 4B).

When the detection of both epithelial (CTCs) and mesenchymal (CMCs) cell types was combined, CTC+CMC frequency in cancer patients was 70%, including 79% of breast and 62% of lung cancer patients, respectively (Fig 3C). This was significantly higher than the 3% detection rate in healthy controls (*p* = 0.001). Mean CTC+CMC levels in breast cancer patients (0.75 cells/mL) were not significantly different from that of lung cancer patients (2.41 cells/mL, *p* = 0.32) (Table 2, Fig 4C).

Putative CSCs were detected in 54% of cancer patients, including 58% of breast cancer patients and 50% of lung cancer patients (Fig 3D). CSC detection in cancer patients was significantly greater (*p*<0.001) than in healthy controls, who did not show any detectable CSCs (Fig 4D).

In marked contrast to CTCs, CMCs, and CSCs, CECs were present in 43% of healthy controls. CECs were detected in 55% of the cancer patients overall, including 53% of the breast cancer and 57% of the lung cancer patients (Fig 3E). CEC detection frequency was not significantly different between cancer patients and controls. Overall, CECs were the most abundant of the cell population studied in cancer patients (mean, 3.61 CEC/ml). Moreover, this mean CEC level in cancer patients was significantly higher than in healthy controls (0.47 CEC/ml, *p* = 0.007), Table 2, Fig 4E).

The mean cell sizes for CMC, CEC, and CSCs were all approximately 10μM in diameter (range, 5 to 15 μM). We did observe recovered cells that were smaller than the pore size. We attribute the ability to trap smaller cells to the cross linking effect of the paraformaldehyde fixation. Moreover, we observed that filtration recovery was the same for all cell types of similar size. Other filtration systems have reported the same recovery for cultured cells of the same size, and reduced recovery for cells with smaller sizes [11, 13, 24, 25].

Other studies have reported the presence of CK-positive and CD45-positive cells in blood of cancer patients [26, 27]; however, these cells were not detected in our samples. We did detect occasional clusters of cells in different cell populations, particularly for CECs (Fig 3F). Putative CSCs, however, were only detected as single cells. No significant difference in the prevalence of cell clusters was observed between lung and breast cancer patients.

### Comparison with the CellSearch^®^ Assay

In 16 duplicate blood samples, CTCs were assayed using both the microfluidic filter-based assay and the CellSearch^**®**^ system. Direct comparison of CTC detection revealed moderate agreement between the two assays (Fig 4F, R^2^ = 0.46; Fig 4G, kappa = 0.47).

### Serial Blood Analysis

In 3 breast cancer patients, serial blood samples were analyzed to evaluate the feasibility of monitoring circulating cell populations over time.

**Fig 5:**
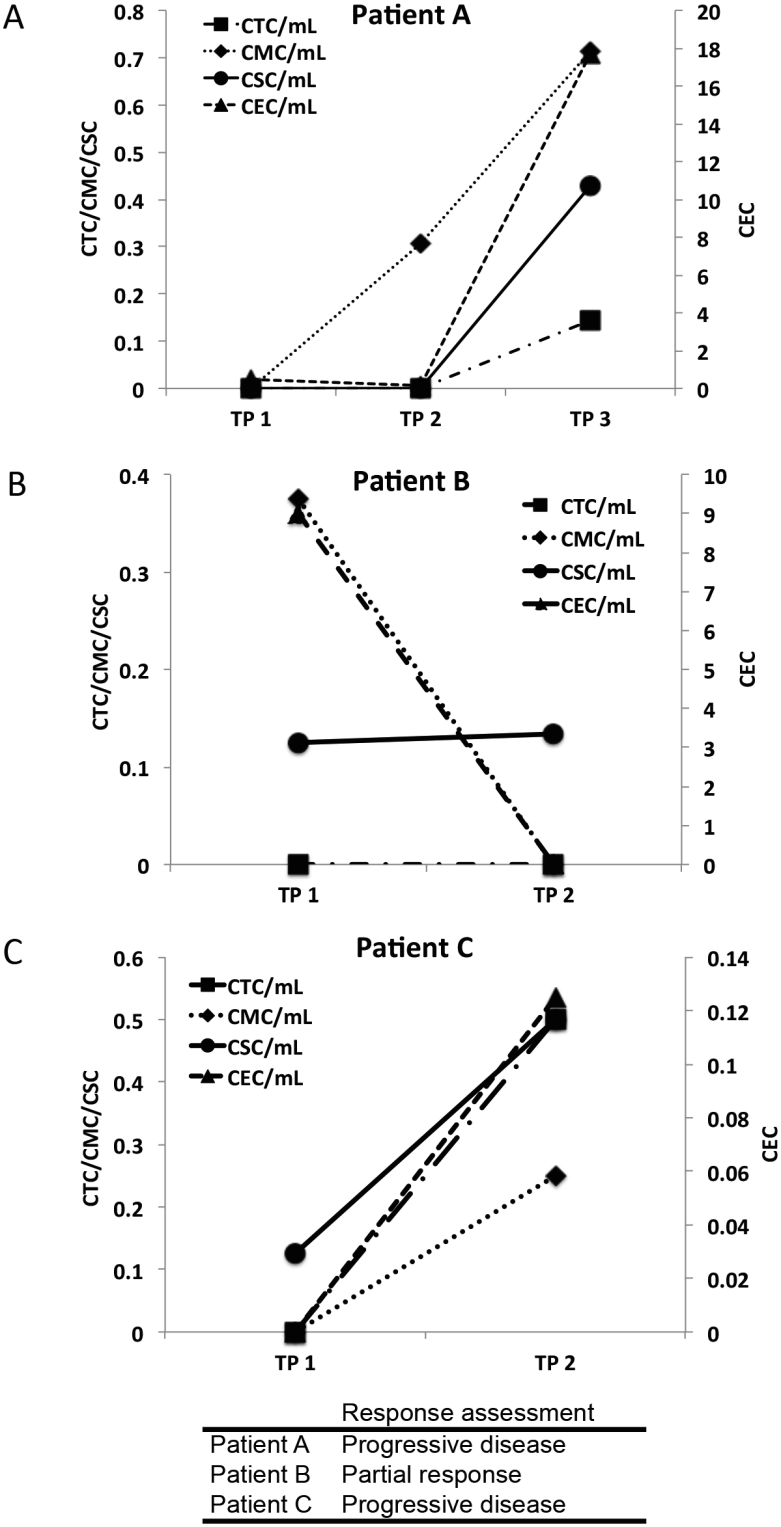
Enumeration of circulating rare cells in serial blood samples from three metastatic breast cancer patients. Circulating tumor cells (CTC), circulating mesenchymal cells (CMC), circulating endothelial cells (CEC), and putative circulating stem cells (CSC) were enumerated in blood samples collected at different time points (TP) during treatment. The y-axis on the left represents the scale for CTC, CMC, and CSC per mL of blood while the y-axis on the right represents the scale for CEC per mL of blood. Clinical response was evaluated using Response Evaluation Criteria in Solid Tumors (RECIST) measurement criteria.

Patient A was a 49-year old woman diagnosed with ER-positive, HER2-negative metastatic breast cancer. Blood samples were collected at days 0, 28, and 84 of a clinical trial (Fig 5A). Microfluidic filter-based analysis revealed a marked increase in all rare cell populations (CTCs, CMCs, CSCs, and CECs) between time points 2 and 3. In contrast, parallel testing via the CellSearch^**®**^ system did not detect any CTCs. Clinical testing for the serum tumor marker CA 15–3 at matching time points revealed a similar increase between time points 2 and 3 (CA 15–3 levels: 84, 88, 124 U/mL). Subsequent clinical assessment confirmed that the patient experienced disease progression.

Patient B was a 54-year old woman diagnosed with ER-positive, HER2-negative metastatic breast cancer. Blood samples were collected on days 0 and 14. Microfluidic filter-based analysis showed no detectable CTCs at either time point. CellSearch^**®**^ was performed on an independent sample approximately three weeks prior to the initial microfluidic filter-based testing (day -21), as well as on a parallel sample at time point 2; these similarly showed no detectable CTCs. However, the levels of CSCs were low and remained unchanged. CMCs and CECs were also detected and levels appeared to decline over time (Fig 5B). Serum CA 15–3 levels assessed 26 days before and 2 days after the initial microfluidic filter-based testing showed a slight decrease (50 and 47 U/mL). Clinical assessment indicated that the patient had a partial response to cancer treatment.

Patient C was a 68-year old woman diagnosed with ER-positive, HER2-negative metastatic breast cancer. Blood samples were collected on days 0 and 30. Microfluidic filter-based analysis showed increased levels of all circulating rare cell populations on day 30 (Fig 5C). CellSearch^**®**^ testing of blood samples collected at identical time points also displayed an increase in CTC levels (7 and 17 CTCs per 7.5mLs of blood). Clinical assessment revealed disease progression.

## Discussion

We developed a novel assay for enrichment, detection and enumeration of circulating rare cell populations in cancer patients. The protocol consists of two key steps: enrichment by filtration using a microfluidic device and sequential multiplex immunostaining to identify rare cell types. After extensive preclinical testing, we evaluated the clinical performance of our assay in detecting and enumerating CTCs, CMCs, CSCs, and CECs in the blood of metastatic breast and lung cancer patients.

A number of microfluidic approaches for isolation of CTCs have been reported [28–32]. For example, the CTC-iCHIP, an automated microfluidic separation system has been used to successfully isolate and analyze CTCs [28, 32]. To our knowledge, none of the existing platforms have been used for multiplex detection of circulating rare cells.

CTCs were frequently detected in breast and lung cancer patients, but were largely absent in healthy controls. The mean level of CTCs was significantly higher in breast compared to lung cancer patients, consistent with previous observations using the CellSearch^**®**^ assay [10]. Head-to-head comparison of our microfluidic filter method vs. the FDA-cleared CellSearch^®^ system revealed moderate agreement.

We have previously reported the feasibility of enumeration and isolation of CTCs by fluorescence-activated cell sorting (FACS) following immunomagnetic enrichment [33–35]. However, both immunomagnetic enrichment and FACS staining were based on EPCAM expression. The filtration-based method used in the current study eliminates the reliance on surface markers for identification of CTCs. Hence, in addition to CTCs with epithelial marker expression, our assay also detected circulating rare cells with mesenchymal marker expression. Indeed, CMCs were detected in 58% of cancer patients, and none were detected in healthy controls. Combined detection of epithelial (CTCs) and mesenchymal (CMCs) rare cell types (i.e., CTC+CMC) was 79% and 62% in breast and lung cancer patients, respectively, representing a potential increase in sensitivity over CTC or CMC alone.

The cancer stem cell hypothesis posits that a rare subpopulation of tumor cells capable of self-renewal and multipotency are responsible for tumor initiation and growth [36, 37]. Several markers, including CD44, CD24, and ALDH1, have been used to detect and enrich for putative CSCs from solid tumors [38–40]. In this study, we used two novel candidate stem cell markers, PIWIL2 [41, 42] and TPGB/5T4 [43, 44], to detect putative CSCs in cancer patients. PIWIL2 is a member of the P-element-induced wimpy testis/Argonaute (PIWI/AGO) family and plays an important role in the development of germ cells [45]. Studies have recently demonstrated that PIWIL2 is expressed in precancerous and cancer stem cells [41, 42, 46, 47]. Additionally, this gene is expressed in different stages of breast cancer but not in normal mammary tissue [48]. The other putative stem cell marker, trophoblast glycoprotein (TPBG/5T4), is an oncofetal protein that is expressed in fetal trophoblasts, the outermost layer of cells in a mammalian embryo [49]. Recent studies have shown that TPBG/5T4 is expressed in tumor-initiating cells in lung cancer [43, 44], and high expression in several cancer types has been associated with inferior clinical outcome [43, 50–52]. In this study, putative CSCs were detected in a majority of breast and lung cancer patients, but were completely absent in healthy controls.

Endothelial cells in circulation may arise from tumor angiogenesis-related processes in cancer patients, as well as from injury of the normal vasculature [53–55]. Changes in CEC levels have been observed in a number of disease settings, including cancer, infections, and cardiovascular disease [56–60]. CECs, however, are frequently observed in disease-free individuals as well [54, 55]. In our study, CECs were the most abundant of the cell populations studied in both cancer patients and in healthy subjects; however, cancer patients did appear to show somewhat higher levels. This finding is consistent with results from a previous study showing that the majority of the circulating rare cells captured by size selection were endothelial cells [19].

To demonstrate the feasibility of serial analysis, circulating rare cells in selected breast cancer patients were assayed at different time points over the course of treatment. Comparison of the numbers of circulating rare cells with the levels of established clinical markers, such as CA 15–3 and CTC assay by Cellsearch^**®**^, showed similar trends. Additionally, preliminary observations in this small number of patients showed that the levels of circulating rare cells may correlate with response outcome.

Although our sequential immunostaining approach ultimately yields highly pure cell populations, cell recovery may further be improved by evaluating different filter and filtration parameters [13, 15]. Also, parallel testing with different isolation techniques, including flow cytometry, may be performed to compare capture efficiencies.

Our approach faces similar limitations as with other size-based methods. For example, it is conceivable that very small circulating rare cells may be able to pass through the filter pores. Nevertheless, the cell recovery efficiency noted here is consistent with other approaches, such as immunomagnetic enrichment/FACS, which is sufficient to provide for detailed molecular analyses by comparative genomic hybridization array [33, 34] and expression array [61].

## Conclusions

Our study demonstrates the feasibility of a novel approach for characterizing multiple rare cell populations in metastatic cancer patients using an automated microfluidic filter device combined with a multiplex immunoassay. CTCs were detected in breast and lung cancer patients, but were largely absent in healthy controls. Detection of CMCs and putative CSCs was achieved using the same platform, and only observed in cancer patients but not controls. Serial blood analysis demonstrated the feasibility of monitoring circulating rare cells over time. In summary, we conclude that simultaneous assessment of multiple circulating rare cell types in cancer patients is technically feasible, and may enable new applications in the study of cancer metastasis and personalized cancer treatment.

## Supporting Information

S1 Fig Sample preparation and staining methods. Flow chart illustrating the blood sample preparation, isolation, and the staining procedures.
(PDF)

S2 Fig CONSORT flow chart. Study flow chart showing the numbers of patients who were enrolled and included in the analysis.
(PDF)

S3 Fig Details of the microfluidic device. A) Top view dimensions of the field of microfluidic posts that reside under membrane in the plastic base. The post field has a diameter of 20.5 mm. The membrane is welded to the plastic base in a ring at 20.5 to 24.0 mm. A ring of through holes resides at a ring diameter of 19. 6 mm so that liquid can be drained in the waste container. B) Side view dimensions of a microfluidic post that the membrane is placed on to. The height is 0.15 mm and the width is 0.30 mm.
(PDF)

S1 File Supplementary methods. Description of reagent preparation, cell isolation, staining and imaging procedures.
(PDF)

S2 File Supplementary information. Optimization of assay parameters.
(PDF)

S1 Table Markers and reagents. List of antibodies and fluorescent labels.
(PDF)
