# Supplementary figures and images for: A Novel Strategy for Detection and Enumeration of Circulating Rare Cell Populations in Metastatic Cancer Patients Using Automated Microfluidic Filtration and Multiplex Immunoassay
Source: PLoS One. 2015 Oct 23;10(10):e0141166. doi: 10.1371/journal.pone.0141166 (PMC4619669; doi:10.1371/journal.pone.0141166)

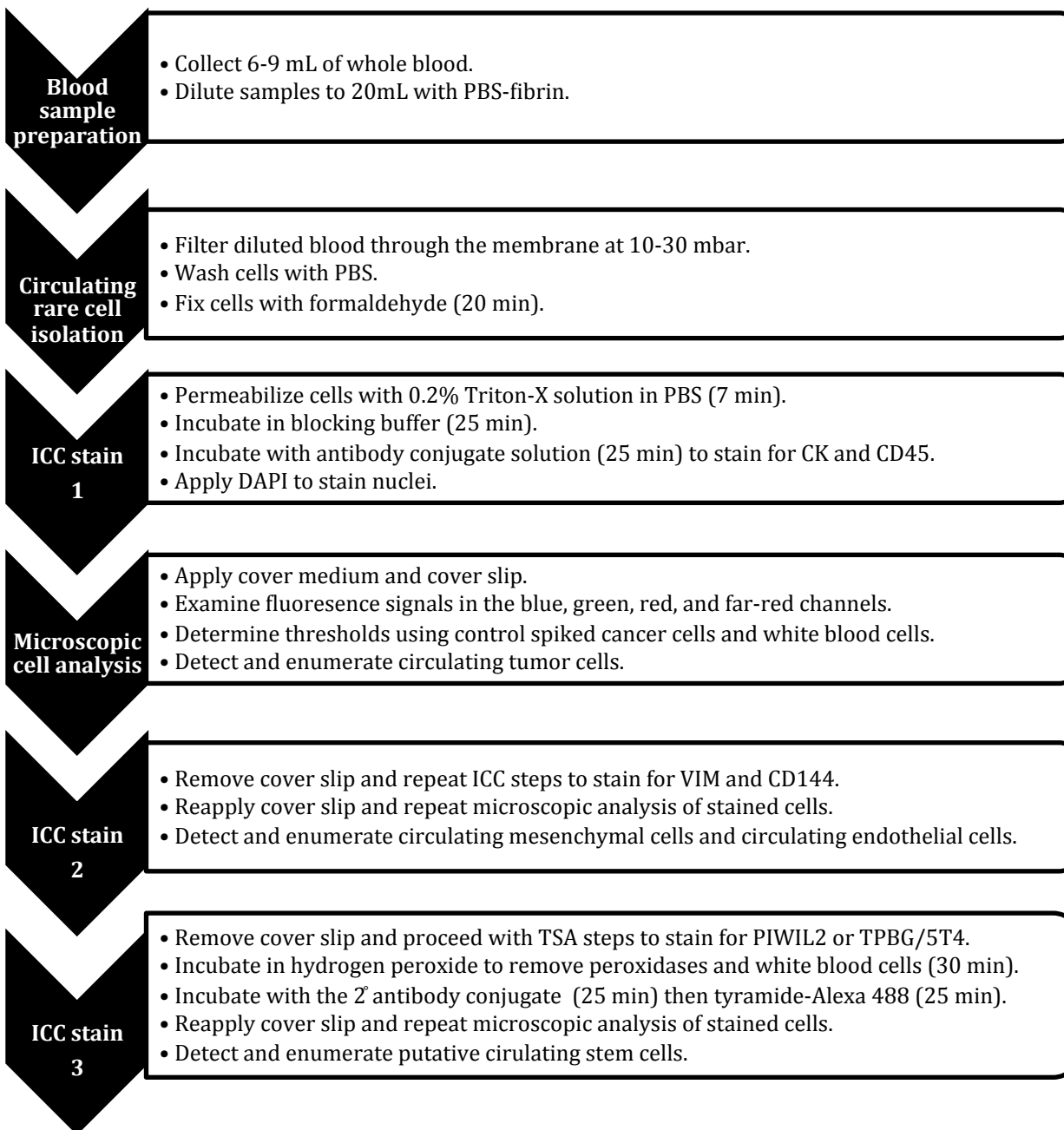

Supplement: S1 Fig — Flow chart illustrating the blood sample preparation, isolation, and the staining procedures. (PDF) [file pone.0141166.s001.pdf]

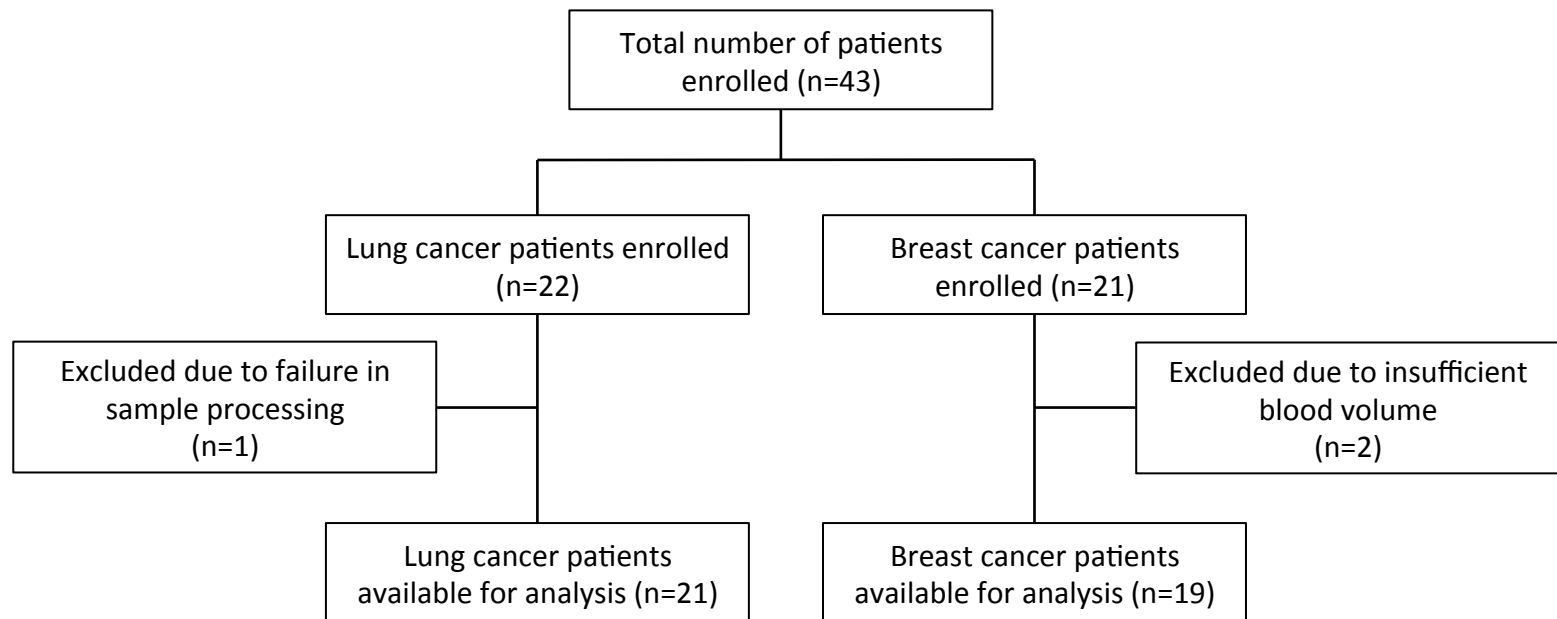

Supplement: S2 Fig — Study flow chart showing the numbers of patients who were enrolled and included in the analysis. (PDF) [file pone.0141166.s002.pdf]

A. Top view

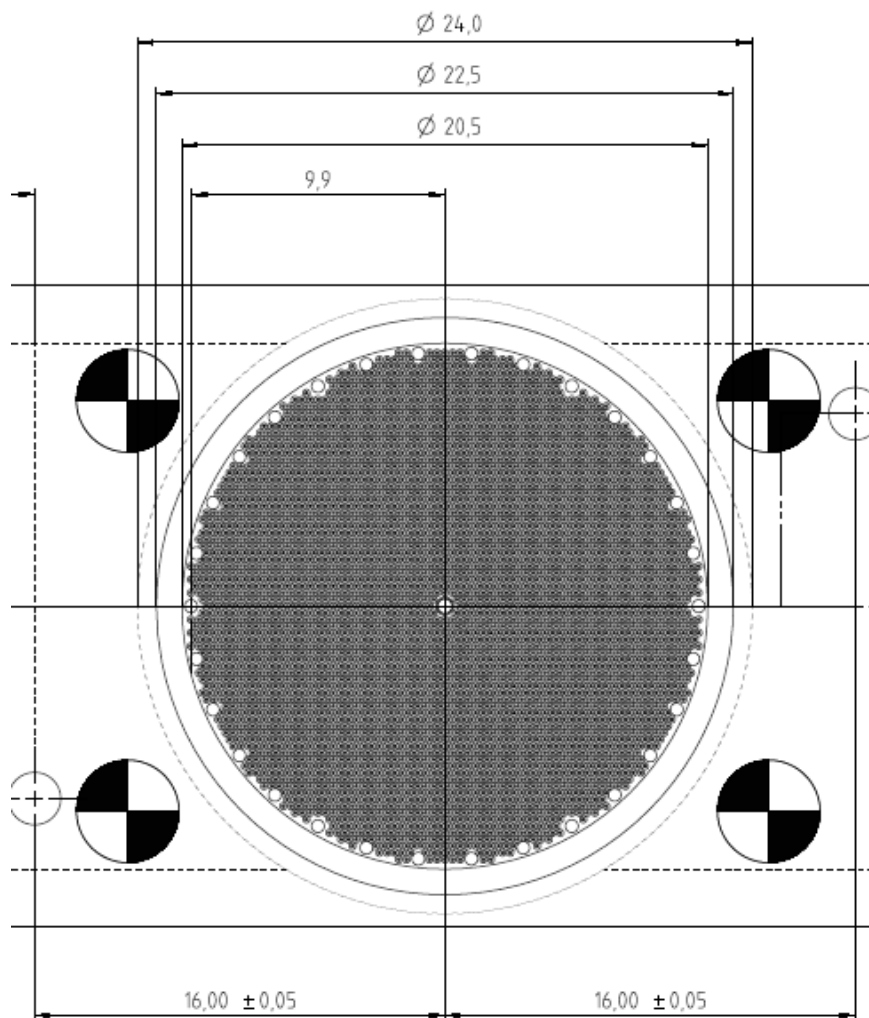

B. Side view

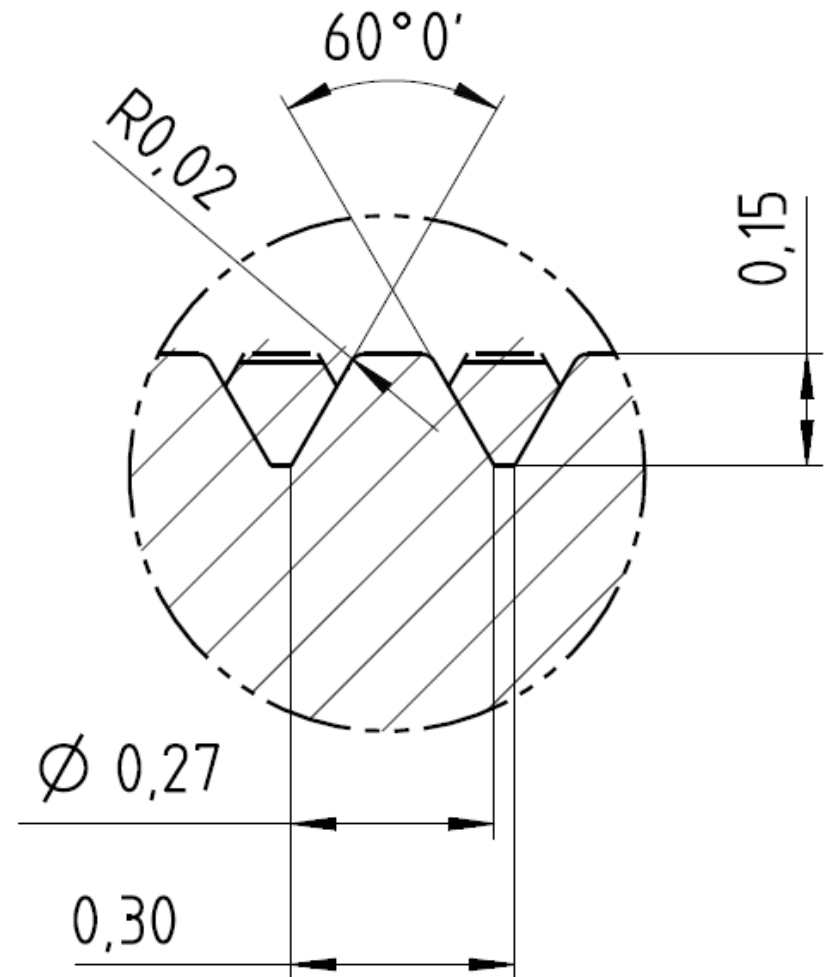

Supplement: S3 Fig — A) Top view dimensions of the field of microfluidic posts that reside under membrane in the plastic base. The post field has a diameter of 20.5 mm. The membrane is welded to the plastic base in a ring at 20.5 to 24.0 mm. A ring of through holes resides at a ring diameter of 19. 6 mm so that liquid can be drained in the waste container. B) Side view dimensions of a microfluidic post that the membrane is placed on to. The height is 0.15 mm and the width is 0.30 mm. (PDF) [file pone.0141166.s003.pdf]
