# Supplementary material for: A Novel Strategy for Detection and Enumeration of Circulating Rare Cell Populations in Metastatic Cancer Patients Using Automated Microfluidic Filtration and Multiplex Immunoassay
Source: PLoS One. 2015 Oct 23;10(10):e0141166. doi: 10.1371/journal.pone.0141166 (PMC4619669; doi:10.1371/journal.pone.0141166)
Supplement: S1 File — Description of reagent preparation, cell isolation, staining and imaging procedures. (PDF) [file pone.0141166.s004.pdf]

Supplementary Methods

Reagent preparation

1. 1x Phosphate buffered solution (1x PBS) was prepared by diluting PBS 10x pH 7.4 (Ambion, part# AM9625) 1:10 in ultrapure deionized water and filtering through a 0.2mm sterile filter.

2. Phosphate buffered solution-Tween (PBS-T) was prepared by adding 0.5mL of Tween®-20 (Promega, part# H5152) to 100mL of PBS 10x pH7.4 and diluting to 1L in ultrapure distilled water. The resulting solution was filtered through a 0.2mm sterile filter.

3. Phosphate buffered solution-fibrin (PBS-fibrin) was prepared in several steps; a solution of 0.1 mg/mL fibrinogen solution was prepared by dissolving 10mg of fibrinogen (Sigma-Aldrich, part# F3879-1G) in 100mL of 1x PBS; a trypsin solution was prepared by dissolving 2.4mg of trypsin (>10,000 BAEE units/mg) in 83mL of 1x PBS; and a benzamidine solution was prepared by dissolving 416 mg of benzamidine (Sigma-Aldrich, part# 434760-5G) in 83mL of 1x PBS and stirring until dissolved. 83mL of PBS 10x pH7.4 was combined with 750mL of ultrapure deionized water and heated in a water bath at 37˚C for 15 minutes. The PBS solution was removed from the water bath, and 833mL of the fibrinogen solution and 83mL of the trypsin solution were added. The resulting solution was heated in the water bath at 37˚C for an additional 5 minutes. The solution was cooled to room temperature, and 83mL of the benzamidine solution was added. The resulting PBS-fibrin solution was filtered through a 0.2mm sterile filter.

4. A 1mg/mL solution of 4',6-diamidino-2-phenylindole (DAPI) or Hoechst was prepared by dissolving solid material in ultrapure deionized water and the resulting solution divided into aliquots of 0.5mL and stored frozen protected from light. The solution was thawed before each use.

5. 1,4-Diazabicyclo [2.2.2] octane (DABCO) cover medium was prepared by adding 0.25mg of DABCO (Sigma-Aldrich, part# D2780-2) to 9mL of glycerol and 1mL of PBS 10x pH 7.4 and gently mixing until dissolved. The solution was divided into aliquots of 0.5mL and stored frozen protected from light. The solution was thawed before each use.

6. Tyramide-Alexa 488 was prepared as directed in the Anti-rabbit Alexa Tyramide Signal Amplification (TSA™) kit (Life Technologies, part# T20922).

7. Permeabilization buffer was prepared by dissolving 400 mL of 10% Triton™ X-100 (Amresco, M236-10mL) in 19.6mL of 1x PBS.

Antibody preparation and signal amplification

The concentration of the primary antibody solution required for detection of cells was based on assay specific protocols. Antibody solutions were added in the required volume of The Blocking Solution (Candor Bioscience, part# 110) in a 15mL Greiner tube.

For the TSA assay, the secondary antibody was diluted in The Blocking Solution in a 1:100 ratio. The TSA assay involves an indirect immunoassay approach where the secondary antibody conjugated to a horseradish peroxidase allows covalent attachment of fluorescent or chromogenic dyes to protein tyrosine groups to amplify signals. The secondary antibodies that were used included a rabbit anti-goat antibody and a goat anti-rabbit antibody to detect primary antibodies bound to Piwi-like protein 2 (PIWIL2) and TPBG/5T4, respectively.

Isolation procedure

Blood samples were transferred into a 50mL Greiner tube (Greiner-Bio-One) and diluted to 20mL with PBS-fibrin (PBS with 0.083% fibrinogen) to increase cell recovery during the filtration process (**Supplementary Figure 1**). Samples were kept at room temperature and processed within 2 hours of dilution.

Circulating rare cell isolation steps were performed using the Hamilton STARlet™ robotic system. A batch of up to 8 samples and controls can be processed simultaneously. After the software was launched, specific information was entered into the program for the downstream procedures to be

completed. During the filtration process, the blood sample on the membrane was subjected to a negative pressure (typically around -10 mbar) depending on the type of reservoir used, sample volume, and filtration rate.

The following are the automated steps for circulating rare cell isolation using the Hamilton STARlet™ robotic system:

1.) 700µL of isopropyl alcohol was pipetted onto the microscope slide to pre-wet the membrane and filtered;
2.) 1mL of PBS was pipetted onto the slide and filtered;
3.) Blood sample was pipetted in 1mL fractions and filtered after each addition until the total 20mL sample was filtered through the slide;
4.) 1mL of PBS was pipetted and filtered; this wash step was repeated 9 more times to wash any remaining blood and materials through the membrane;
5.) 1mL of 4% formaldehyde in PBS was pipetted and filtered;
6.) 1mL of 4% formaldehyde in PBS was incubated on the membrane for 20 minutes before filtering;
7.) 1mL of PBS was pipetted and filtered to wash the membrane (repeated once).

Immunocytochemistry (ICC) staining procedure

The following are the automated ICC staining and re-staining steps using Hamilton STARlet™ robotic system:

1.) 1mL of 0.2% TritonX in PBS was pipetted and filtered;
2.) 1mL of 0.2% TritonX in PBS was pipetted and incubated for 7 minutes and filtered;
3.) 1mL of PBS was pipetted and filtered, and repeated 4 more times;
4.) 900µL of 3% hydrogen peroxide was pipetted in PBS and incubated for 30 minutes (only for TSA re-stain procedure);
5.) 1mL of PBS was pipetted and filtered (repeated 4 more times for TSA re-stain procedure);
6.) 1mL of The Blocking Solution (Candor Bioscience, part # 110) was pipetted and incubated for 25 minutes before filtering;
7.) 1mL of PBS-T (0.05% Tween in PBS) was pipetted and filtered (repeated once);
8.) 260mL of the antibody solution (300mL for re-stain procedure) was pipetted and incubated for 25 minutes;
10.) 1mL of PBS-T was pipetted and filtered (repeated 4 more times);
11.) For an indirect TSA immunoassay method, 260mL of a 10mg/mL solution of the secondary antibody was pipetted and incubated for 25 minutes before filtering; 1mL of PBS-T was pipetted and filtered (repeated 4 more times)
12.) 260mL of tyramide-Alexa 488 was pipetted and incubated for 25 minutes before filtering;
13.) 1mL of PBS-T was pipetted and filtered (repeated 4 more times);
14.) 500mL of 4',6-diamidino-2-phenylindole (DAPI) was pipetted and incubated for 1 minute;
15.) 1mL of PBS-T was pipetted and filtered (repeated once).

Microscopic analysis

Once the robotic method was completed, the microscope slides with membranes containing the isolated and stained cells were removed from the filtration unit, and the bottoms of the slides were each wiped dry with a clean Kimwipe. A 23mL drop of 1,4-Diazabicyclo [2.2.2] octane (DABCO) cover medium was placed on the membrane, and a 22mm diameter cover slip was carefully placed over the top.

When re-staining cells on a membrane, the slide cover was carefully removed with tweezers, and the slide was remounted onto the Hamilton STARlet™ robot. Before proceeding through the robotic ICC staining steps described above, the membrane was washed with 1mL of PBS and filtered to remove DABCO cover medium.
