# Supplementary material for: A Novel Strategy for Detection and Enumeration of Circulating Rare Cell Populations in Metastatic Cancer Patients Using Automated Microfluidic Filtration and Multiplex Immunoassay
Source: PLoS One. 2015 Oct 23;10(10):e0141166. doi: 10.1371/journal.pone.0141166 (PMC4619669; doi:10.1371/journal.pone.0141166)
Supplement: S2 File — Optimization of assay parameters. (PDF) [file pone.0141166.s005.pdf]

Supplementary information

Optimization of the fixation process

The fixation procedure for freshly drawn whole blood was initially optimized by evaluating different concentrations of fixative (paraformaldehyde). We found that the addition of high concentrations of paraformaldehyde (over-fixation) produced cell clumps that clogged the filter, while too little paraformaldehyde (under-fixation) resulted in decreased cell recovery rates. Indeed, observations using spike-in experiments showed that unfixed cultured cancer cells can be pliable and can pass through the 8μM pores of the filter membrane. Also, unfixed cells were difficult to image, as cell morphology were typically not left intact after the filtration process. Ultimately, the use of Transfix® (Vacutest Kima), a commercially available fixative that contains 1% paraformaldehyde, resulted in decreased cell clumping (less clogs), increased yield and improved imaging results. Further testing revealed that fixed blood samples could be stored at ambient laboratory temperature and were stable up to 60 hours.

Optimization of the filtration process

The filtration process involved the application of alternating negative and positive pressures. Previous observations have shown that applying high vacuum pressures (negative pressures >30 mbar) can negatively impact cell recovery rates [13], while lower vacuum pressures (negative pressures <30 mbar), as expected, increased processing times. We found that the application of oscillating negative pressure of ≤30 mbar and positive pressure of 10 mbar to achieve a flow rate of approximately 1mL/min significantly improved recovery rates in spike-in experiments. Also, applying a non-continuous vacuum prevented the buildup of cells near the pores, which in turn prevented the filter from clogging.

Optimization of the washing process

To facilitate the removal of non-target cells (e.g., red blood cells) and excess reagents (e.g., unbound antibodies) during the washing steps, the filter membrane was placed on top of microfluidic posts that were molded onto the plastic slide base (**S3 Fig.**). These microfluidic structures allowed for efficient removal of flow-through (i.e., wash solutions) while supporting the membrane during the washing procedure.
