# Supplementary material for: A Novel Strategy for Detection and Enumeration of Circulating Rare Cell Populations in Metastatic Cancer Patients Using Automated Microfluidic Filtration and Multiplex Immunoassay
Source: PLoS One. 2015 Oct 23;10(10):e0141166. doi: 10.1371/journal.pone.0141166 (PMC4619669; doi:10.1371/journal.pone.0141166)
Supplement: S1 Table — List of antibodies and fluorescent labels. (PDF) [file pone.0141166.s006.pdf]

Marker	Clone	Vendor	Fluorescent probe	Emission Color	Excitation filter	Emission Filter
Nucleus	n.a.	n.a.	DAPI	Blue	360/40	470/40
CK8/18	UCD/PR10.11	Siemens	DyLight550	Red	546/12	600/40
CK19	A53-B/A2	Siemens	DyLight550	Red	546/12	600/40
PanCK	AE1/AE3	Ebioscience	Fluor570	Red	546/12	600/40
CD45	9.5	Siemens	DyLight650	Far-red	620/60	700/75
CD144	FB	Siemens	DyLight488	Green	480/40	527/30
Vimentin	V9	Siemens	DyLight550	Red	546/12	600/40
PIWIL-2	K-18	Santa Cruz Biotech	Alexa 488	Green	480/40	527/30
TPBG/5T4	EPR5530	AbCam	Alexa 488	Green	480/40	527/30
